# Intra-Articular Injection of Platelet-Rich Plasma Is More Effective than Hyaluronic Acid or Steroid Injection in the Treatment of Mild to Moderate Knee Osteoarthritis: A Prospective, Randomized, Triple-Parallel Clinical Trial

**DOI:** 10.3390/biomedicines10050991

**Published:** 2022-04-25

**Authors:** Dawid Szwedowski, Ali Mobasheri, Andrzej Moniuszko, Jan Zabrzyński, Sławomir Jeka

**Affiliations:** 1 Orthopedic Arthroscopic Surgery International (O.A.S.I.) Bioresearch Foundation, Gobbi N.P.O., 20133 Milan, Italy; 2 Department of Orthopaedics and Trauma Surgery, Provincial Polyclinical Hospital, 87-100 Torun, Poland; 3 Research Unit of Medical Imaging, Physics and Technology, Faculty of Medicine, University of Oulu, FI-90014 Oulu, Finland; ali.mobasheri@oulu.fi; 4 Department of Regenerative Medicine, State Research Institute Centre for Innovative Medicine, Santariskiu 5, LT-08406 Vilnius, Lithuania; 5 Departments of Orthopedics, Rheumatology and Clinical Immunology, University Medical Center Utrecht, 3508 GA Utrecht, The Netherlands; 6 Department of Joint Surgery, The First Affiliated Hospital, Sun Yat-sen University, Guangzhou 510080, China; 7 Department of Internal Medicine and Gastroenterology with Inflammatory Bowel Diseases Unit, Central Clinical Hospital of the Ministry of the Interior and Administration, 02-507 Warsaw, Poland; moniuszko.a@gmail.com; 8 Department of General Orthopedics, Musculoskeletal Oncology and Trauma Surgery, University of Medical Sciences, 61-701 Poznan, Poland; zabrzynski@gmail.com; 9 Department and Clinic of Rheumatology and Connective Tissue Diseases, University Hospital No. 2, Collegium Medicum UMK, 85-168 Bydgoszcz, Poland; s.jeka@wp.pl

**Keywords:** knee osteoarthritis, injections, intra-articular, osteoarthritis, platelet-rich plasma, viscosupplementation, glucocorticosteroids

## Abstract

Purpose: To prospectively compare the efficacy and safety of intra-articular injections of platelet-rich plasma (PRP) with hyaluronic acid (HA) and glucocorticosteroid (CS) control groups for knee osteoarthritis (KOA) in a randomized, triple-parallel, single-center clinical trial. Methods: A total of 75 patients were randomly assigned to one of three groups receiving a single injection of either leukocyte-poor platelet-rich plasma (25 knees), hyaluronic acid (25 knees), or glucocorticosteroid (25 knees). The Western Ontario and McMaster Universities Osteoarthritis Index (WOMAC) score was collected at baseline and 6, 12, and 26 weeks after treatment. Results: After 6 weeks of PRP administration, a decrease in the mean WOMAC value was observed in all three study groups. Three months after administration, the greatest decrease in the mean WOMAC value was obtained in the PRP group. The results in the HA and CS groups were similar (*p* = 0.681). In the one-way analysis of variance and post hoc analysis using the HSD Tukey test, a significantly greater improvement was shown by comparing the PRP and CS groups (*p* = 0.001), and the PRP and HA groups (*p* = 0.010). After intra-articular injection of CS, the reduction in pain was greatest 6 weeks after administration, and the mean value was the lowest among all groups. During subsequent visits, the value of the pain subscale increased, and after 6 months, it was the highest among the studied groups. Using the Wilcoxon paired test, no PRP effect was found to reduce stiffness at the 6-month follow-up (*p* = 0.908). Functional improvement was achieved in all groups, i.e., a decrease in the value of this subscale 6 months after administration. The largest decrease was seen in the group that received PRP (*p* < 0.001) and then in the HA group. The smallest decrease among the investigated methods was shown in the CS group. Conclusions: Intra-articular injections of PRP can provide clinically significant functional improvement for at least 6 months in patients with mild to moderate KOA which is superior to HA or CS injections.

## 1. Introduction

Osteoarthritis (OA), which is the most common disease of the joints, is a significant social and economic problem, especially in an aging society [1]. The pathogenesis of OA is multifactorial [2]. The molecular basis of degenerative changes is becoming better known thanks to numerous biochemical and genetic studies [3]. In recent years, intra-articular injections have become more and more popular in the moderate therapy of knee osteoarthritis (KOA). Due to the complexity of the processes in the knee joint with OA, all the mechanisms responsible for joint tissue degeneration are not understood yet and the disease-modifying drugs are still missing. Therefore, the main goal of OA therapy is to relieve pain and improve the function of the knee joint. Platelet Rich Plasma (PRP) is an autologous solution of highly concentrated platelets dispersed in a small volume of plasma, containing platelet growth factors. Growth factors promote proliferation and angiogenesis, reducing critical inflammatory regulators and the expression of inflammatory enzymes [4,5,6]. It is a relatively new method of intra-articular treatment. It is a low invasive one-step procedure treatment with minimal risk of adverse reactions. In most cases, PRP therapy can be used as a solo procedure. However, despite the growing popularity of this therapy, there is still a lack of randomized studies showing greater effectiveness of this method over other treatments. The aim of this study was not only to assess the effectiveness in relieving pain and improving functions but also the safety of using intra-articular injections with PRP in OA in a 6-month follow-up period. An attempt was made to answer the question of whether the clinical improvement after using this method of treatment is significantly higher than after the injections of hyaluronic acid or glucocorticoid.

## 2. Methods

### 2.1. Patient Selection and Screening

This was a parallel-group, trial with equal randomization. The study protocol was approved by an appropriate Institutional Review Board and was publicly accessible before the enrolment of the first patient. We performed the study in accordance with the ethical standards outlined in the 2013 revision of the 1975 Declaration of Helsinki, and we report the results according to the 2010 CONSORT statement. The written consent signed by each participant included the methods for PRP, HA, and CS injection in this trial, as well as benefits and possible adverse effects.

Eligible patients had radiographic evidence of osteoarthritis (weight-bearing views) assessed as Kellgren–Lawrence grade 2 (definite osteophytes and possible joint space narrowing) to grade 3 (moderate multiple osteophytes, definite narrowing of joint space and some sclerosis and possible deformity of bone ends) and met the criteria of the European League Against Rheumatism (EULAR), which define the criteria for the diagnosis of osteoarthritis of the knee joints [7]. We excluded patients who had received an intra-articular injection in the previous 12 months. All participants were assessed with a WOMAC outcome questionnaire (each with a score of 0–100). Detailed inclusion and exclusion criteria are provided in Table 1.

The subject screening was performed in the outpatient department, where the author (D.S.) evaluated patients’ eligibility for study inclusion through history taking, physical examination, laboratory testing, and imaging studies. The K-L stage of OA was independently assessed by the senior author (S.J.) Nonsteroidal anti-inflammatory drugs and chondroprotective supplements were prohibited from being taken during the duration of the trial. Paracetamol was permitted during the study but had to be discontinued 48 hours before each follow-up assessment.

#### Randomization

Subjects who met all the inclusion criteria were randomly assigned at a 1:1:1 ratio to 1 of 3 treatment groups to receive intra-articular injection: group 1, PRP (Density Platelet Gel, IBF, Scafati, Italy); group 2, HA (Biovisc Ortho Single, 30 mg/mL, molecular weight 3.400–3.800 kDa, Atradis Medical Devices, Warsaw, Poland) and group 3, CS (Diprophos, 6.43 mg/mL betamethasone dipropionate and 2.63 mg/mL betamethasone sodium phosphate, MSD, Warsaw, Poland) through a computer-generated randomization system.

### 2.2. Intervention Protocol

All intra-articular injections were performed through a lateral mid-patellar approach aseptically by the first author (D.S.). Local anesthesia was not used during the intervention to avoid changing the pH of the intra-articular microenvironment. The procedures during the control visits were carried out in the following order:

1. History of ailments and medications taken.
2. Completing the WOMAC questionnaire.
3. Physical examination.
4. Information on further proceedings.
5. Preparation of PRP.

To prepare PRP, a small amount of peripheral blood (10–12 mL) was collected and then placed in a special centrifuge (Zenithlab 80-2C, Zenith Lab Inc., Pomona, CA, USA). During centrifugation, the plasma fraction was separated from the rest of the whole peripheral blood, thus concentrating the platelets and obtaining 7–8 mL of PRP. The PRP centrifuge was set to 4000 rpm and had a duration of 5 min.

### 2.3. Statistical Methods

To determine an adequate sample size for the study, we performed a power analysis using free software (G*Power). A minimum total sample size requirement of 57 knees (or 19 knees per treatment arm) was calculated based on a study power of 80% (β 0.02), a false-positive rate of 5% (α 0.05), and effect size (Cohen f) of 0.15. This study was then designed to enroll approximately 25 knees per group at baseline in anticipation of a possible dropout rate of 10%.

A total of 73 patients who completed the study were statistically analyzed and further analyzed by subgroups. Statistical calculations were performed using the statistical program SPSS Statistics V27, 2020 (Chicago, IL, USA). Descriptive statistics-median and 25th and 75th percentiles or mean values with standard deviation (SD, ±) and a range, depending on the type of distribution were used to analyze the material. A Pair-of-observation Wilcoxon test was used to assess the efficacy of different treatments at randomization and the end of the follow-up period. One-way analysis of variance (ANOVA) was used to rule out subgroup heterogeneity at randomization and to assess the differences in the effectiveness of the 3 types of therapy in individual time intervals, along with post hoc analysis with the Tukey HSD test. Multivariate linear regression analysis was used to exclude the potential influence of gender, BMI, and age on endpoints. Statistical significance was set at *p* < 0.05 throughout. 

## 3. Results

**Figure 1:**
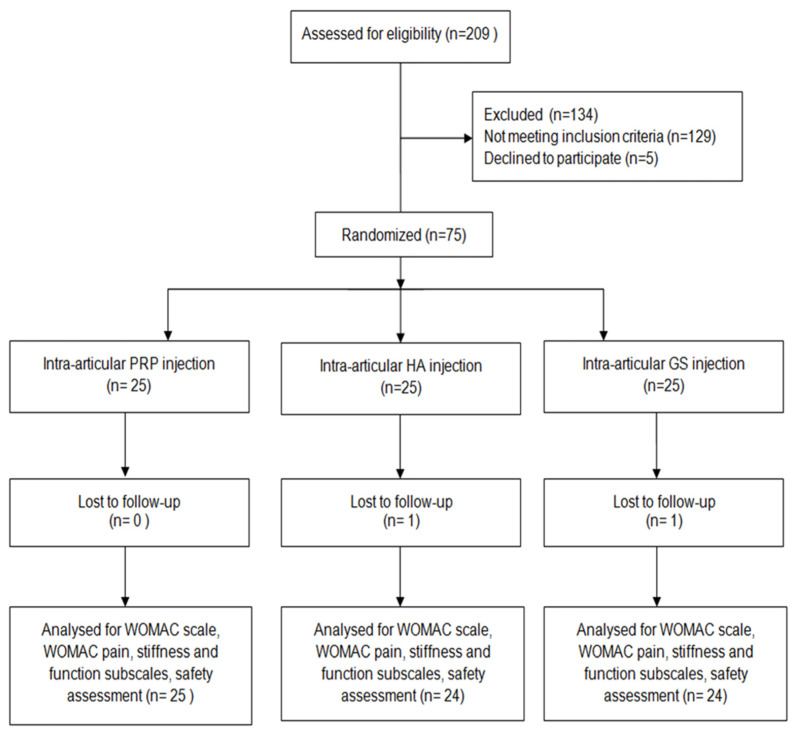
The CONSORT flow diagram of the trial.

From April 2019 to March 2020, a total of 209 patients were assessed for eligibility. Of these patients, 129 did not meet the inclusion criteria and 5 declined to participate. The study included 75 patients, 25 in each group. Seventy-three patients completed the study, as 2 patients (one patient with HA and one patient in the CS group) did not show up for follow-up interviews. The flow diagram of the trial is presented in Figure 1. The mean age of the group in the PRP group was 58 ± 10 years, while in the HA and CS groups, it was 53 ± 7 years and 57 ± 8 years, respectively. The gender distribution was presented in Supplementary Material (Supplementary Figure S1).

There were no significant demographic differences among the 3 groups across sex proportion, age, BMI, and K-L stage for OA, as well as pretreatment WOMAC score (Table 2). No serious adverse effects related to the intraarticular injection were reported among the 3 groups.

**Figure 2:**
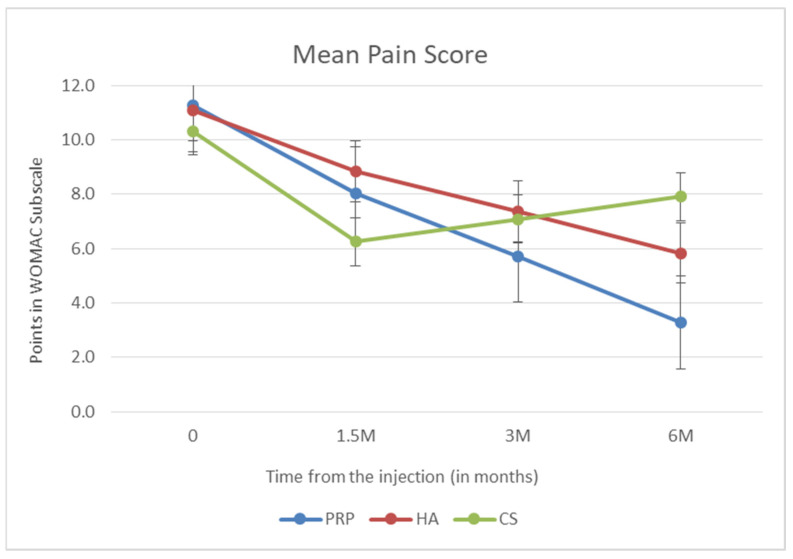
Mean pain score (points in WOMAC subscale).

In the first stage of the analysis, the effectiveness measured on the WOMAC scale was assessed for each of the administered injections after 6 weeks (1.5 months), 3 months, and 6 months after the injection. The first subscale examined in the WOMAC scale is the assessment of pain in the knee joint. It is the most important part because pain is the most common reason for medical consultations and treatment. In the PRP group and the HA group, a linear decrease in the pain value of the WOMAC subscale was demonstrated during subsequent follow-up visits. In the PRP group, the decrease was greater (*p* < 0.001). After intra-knee glucocorticosteroids, the reduction in pain was greatest 6 weeks after administration, and the mean value was the lowest among all groups. During subsequent visits, the value of the pain subscale increased, and after 6 months, it was the highest among the studied groups. The above data are presented in Figure 2.

**Figure 3:**
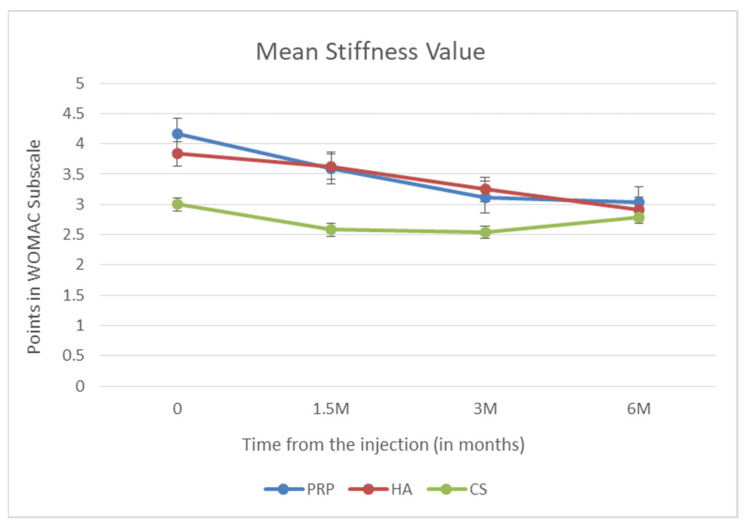
Mean stiffness value (points in WOMAC subscale).

Another WOMAC subscale is the stiffness rating. Using the Wilcoxon paired test, no drug effect was found to reduce stiffness at month 6 after dosing (*p* = 0.908). Changes in the mean stiffness are presented in Figure 3.

**Figure 4:**
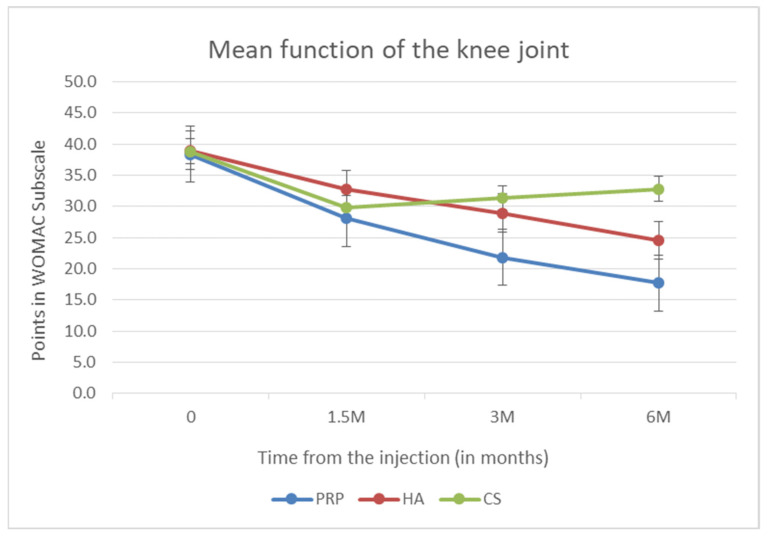
Mean function of the knee joint (points in WOMAC subscale).

The last and most extensive subscale of the WOMAC scale is the evaluation of the knee joint function. Functional improvement was achieved in all groups. The greatest decrease was seen in the group that received PRP (*p* < 0.001), and then, in the HA group. The smallest decrease among the tested methods was shown in the CS group. In the latter group, analogically to the pain subscale, the greatest decrease was visible 6 weeks after administration. Then, during subsequent visits, the subscale values increased, indicating a deterioration of the joint function. The above data are presented in Figure 4.

**Figure 5:**
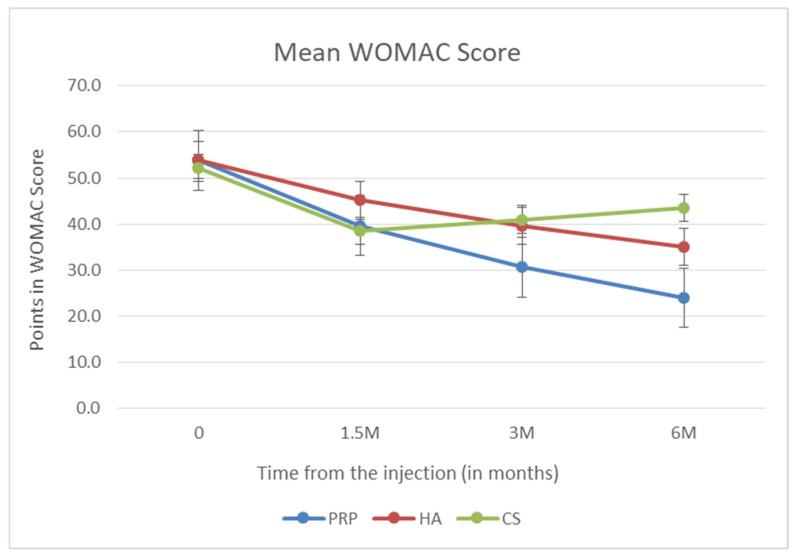
Mean WOMAC value.

The most important and primary endpoint of the study was the analysis of the mean WOMAC value 6 months after dosing. The ANOVA test showed statistically significantly lower results in the PRP group than in the other groups. The statistical significance was, respectively, *p* = 0.002 for PRP versus HA and *p* < 0.001 for PRP versus CS. Patients who received HA obtained a lower mean WOMAC score than patients in the CS group, and the difference between these values was also statistically significant (*p* = 0.006). The above data are presented in Figure 5.

**Figure 6:**
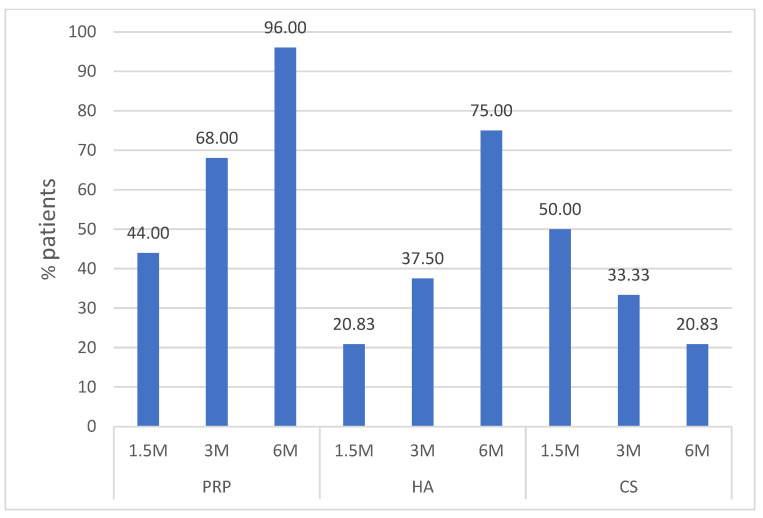
Clinically important improvement.

The final stage of the analysis of the results was to check which proportion of patients (expressed as a percentage) achieved a significant improvement. i.e., after 6 months, there was a reduction in knee pain measured on the WOMAC scale by at least 25% compared to the baseline values. Figure 6 shows the percentage of patients who significantly improved at a given time point (6 weeks and 3 and 6 months after treatment). The Chi-square test showed statistically significant differences for this parameter both between PRP and HA (*p* = 0.038) and between PRP and CS (*p* < 0.001).

### Analysis of the Safety Profile of Individual Methods

In the PRP group, one complication of nausea and dizziness occurred immediately after administration of the injection in a patient with a history of similar complaints during blood collection in the past. After a few hours, the symptoms subsided. In the hyaluronic acid group, one patient reported a headache several hours after intra-articular administration. The pain subsided on the same day without the need for pain medication. The group of patients who received glucocorticosteroid developed a complication in the form of pain and redness at the injection site, which persisted for several hours after administration. There were no serious advertisements (SAEs) in any of the groups.

## 4. Discussion

Most often in the literature, the effectiveness of PRP in the treatment of OA was compared with that of hyaluronic acid [8,9,10,11]. Although several randomized trials have been conducted, this is still a matter of controversy. The results of these studies vary considerably, and so far, no consensus has been reached regarding the higher efficacy of PRP compared to hyaluronic acid. In 2015, Filardo et al. [12] published the results of a randomized trial that enrolled 192 patients with moderate degenerative changes in the knee joints. After one year of follow-up, clinical improvement was demonstrated in both the PRP and hyaluronic acid groups. However, there was no statistically significant difference between the results in both groups. In 2019, the results of a 5-year follow-up of the same group of patients were published [10]. This study also showed functional improvement in both groups and no statistically significant difference between the groups. Cole et al. [8] obtained similar clinical results assessed in the WOMAC scale in a study on a group of 111 people. Additionally, they investigated the effect of platelet-rich plasma and hyaluronic acid on pro-inflammatory and anti-inflammatory markers in synovial fluid. A decrease in the activity of IL-1β and TNF-α was observed in the knee joint after administration of PRP. This finding suggests that the anti-inflammatory properties of PRP may contribute to reducing the symptoms of OA. The above-mentioned studies suggest no statistically significant difference in the effectiveness of PRP compared to HA.

However, several studies have been recently published with opposite results. Görmeli et al. [13] in a study comparing the effectiveness of PRP, hyaluronic acid, and placebo in the form of saline, showed the superiority of PRP over other methods. They enrolled 168 patients in the study, dividing them into two groups according to the stage of advancement assessed on X-rays in the K-L scale: early changes (KL I-III) and advanced changes (K-L IV). In each of the subgroups, a statistically significant advantage of PRP in the improvement of the knee joint function, and pain relief was demonstrated. Similar results were published in 2016 by Duymus et al. [14] assessing the improvement after injection of PRP, HA, and ozone therapy. Platelet-rich plasma showed significantly higher efficacy in relieving OA symptoms than viscosupplementation and ozone therapy. Lana et al. [15] compared the function and pain after using PRP, hyaluronic acid, and a combination of hyaluronic acid and PRP. In a multicenter study, they obtained better WOMAC scores in the PRP group than in the HA group. However, there was no difference between PRP and the combination of PRP with hyaluronic acid in one year of follow-up. In the meta-analysis published in 2020, Belk et al. [11] presented the results of 18 randomized clinical trials comparing the effectiveness of PRP and hyaluronic acid in relieving the knee discomfort associated with OA. The meta-analysis included 811 patients, and most of the studies included in it had a 6-month follow-up period. The basic scale for assessing pain and function of the knee joint was the WOMAC scale. The study showed statistically significantly better functional results after administration of PRP than after administration of hyaluronic acid, which was also observed in the present study.

In the meta-analyses and randomized clinical trials published so far, very few of them included a control group in the form of CS injections, despite their widespread use. Thus, our work presents three commonly used intra-articular treatments for OA analyzed multivariate. Strict inclusion and exclusion criteria made it possible to select patients with moderate degenerative changes in whom intra-articular injections have the greatest chance of improving the patient’s health [16]. Obese patients, in whom the use of intra-articular injection is of secondary importance in changing the course of OA concerning weight reduction, were excluded from the study [17]. Among the patients included in the study—with a BMI below 40 kg/m^2^, both this parameter, age, and gender did not affect the results measured on the WOMAC scale. Placebo in the form of saline injections was not included in this study due to the lack of improvement reported in the literature and significantly worse results on clinical scales compared to PRP [18]. Patel et al. conducted a randomized, double-blind clinical trial comparing single and double injection of PRP, and placebo [19]. It is also interesting that there was no statistically significant difference between the group receiving one injection and the group receiving two injections of PRP.

As already mentioned in most of the studies conducted on intra-articular injections in the treatment of OA, patients were most often assessed using the WOMAC scale during a 6-month follow-up period [11,16]. This is the generally accepted period after which a possible decision to re-inject should be made. It is probably related to the popularity of intra-articular CS injections. As shown in the results presented in this study, the effectiveness of this method is the highest in the 6th week after administration, but only 20.8% of patients feel a significant improvement after 6 months. For comparison, in the PRP group, a significant clinical improvement was achieved in 96% of patients at this endpoint. The results of this study suggest that in the case of PRP, the interval between injections can be extended, as the vast majority of patients receiving PRP do not require another administration after 6 months. CS injections are still widely used [20]. Therefore, this method was also used in the control group of this PRP efficacy study. An article with data from the Humana database of over a million patients treated for OA in 2007–2015 showed that 38% of patients had received an intra-articular GC injection [21]. Although complications following CS administration in the form of accelerated cartilage degradation, joint infections, or subchondral fracture are rare, they are serious and can significantly worsen the course of OA [22]. Therefore, the results presented in this study, showing a short-term and much lower efficacy compared to hyaluronic acid and platelet-rich plasma, may change the treatment regimens and guidelines that still recommend intra-articular GC injection as one of the basic methods of treatment of OA [23].

One of the reasons for the planned single injection into the knee joint was the work of Nakazawa et al. showing the toxicity of serial intraarticular administration of CS on articular cartilage [24]. In addition, some of the studies published so far have assessed the effectiveness of multiple administrations of PRP with multiple administrations of HA, which makes it difficult to evaluate the results and create meta-analyses and may also affect the popularization of administration regimens that may only have a marketing purpose. The subject of platelet-rich plasma efficacy assessment is controversial as there is a lack of standardization. The current classifications and systems for obtaining PRP do not consider the number of growth factors contained nor the influence of individual factors on the course of OA because the performance of such tests in each patient receiving PRP is very expensive. In addition, the multitude of available systems for obtaining PRP and the variety of hyaluronic acids make it difficult to compare the results between studies. It should also be remembered that other factors, such as drug cost and patient preferences, may play a large role in the treatment of patients with OA [25]. In addition, longer (5–10 years) observation periods will answer the question of whether PRP can only minimize the symptoms of OA or also prevent or slow down the progression of OA. With more research on PRP in OA and the growing popularity of this method, it may turn out that the intra-articular injection of PRP in the early stage of OA will reduce the financial costs associated with this disease, including the need for total knee replacement surgery. For this purpose, it is necessary to conduct high-quality multicenter clinical trials on a large population, which in addition to assessing the effectiveness and safety will provide information on the cost-effectiveness and cost-safety of this treatment method for patients and the health system.

## 5. Limitations

First, the study was not blinded, with the risk of reducing the reliability of the results. Both the researcher and the respondent knew what method would be administered to the knee joint. Blinding would require blood collection also in the control groups. The collected blood samples would not be used but would have to be disposed of. Therefore this procedure was abandoned. Secondly. the number of people included in the study (*n* = 75) seems to be smaller than in some other trials on a similar topic. However, usually, most enrolled subjects had bilateral knees participated which is associated with an increased risk of bias, Due to the extensive inclusion and exclusion criteria for this study. and including patients with only one knee affected by OA, our study design closely reflects the actual clinical practice and reduced the risk of bias.

## 6. Conclusions

The encouraging results of this study assessing the efficacy and safety of PRP in OA may in the future contribute to multicenter studies involving the standardization and optimization of PRP levels and to the initiation of studies using this method in OA of other joints. An increasing number of studies with appropriately designed inclusion and exclusion criteria, as well as with the use of modern methods of molecular biology assessing, for example, pro-inflammatory markers in synovial fluid, may contribute to the development of the so-called personalized medicine, i.e., choosing the right treatment for the right patient with knee OA.

## Figures and Tables

**Table 1:** Inclusion and exclusion criteria.

**Inclusion Criteria**
- Diagnosis of OA changes stage II-III Kellgren–Lawrence by radiography (postural bilateral lower limb, weight-bearing standard knee anterior-posterior view in full extension, lateral view in 30 of flexion). - No prior PRP injection in the knee. - No prior surgical procedure in participating knee. - Unilateral Visual Analog Scale (VAS) pain score 4–9 for >6 months. - Age between 40 and 75 years. - BMI < 40 kg/m^2^ - Ability to provide informed consent. - Conservative treatment in the form of exercise, weight reduction, and physical therapy for at least 6 months, without improving the function and reducing pain. - The patient will refrain from treating the knee joints by intra-articular injection, surgery, and physical therapy for the duration of the study. - The patient refrains from taking pain relievers, including non-steroidal anti-inflammatory drugs (NSAIDs), for the duration of the study. Only paracetamol preparations are allowed on an ad hoc basis with a ban on taking them 24 h before the visit.
**Exclusion Criteria**
- Type II diabetes. - Fibromyalgia. - Anemia. - Coagulation disorders or taking anticoagulants. - Pregnancy or lactation. - Allergic to a steroid drug or hyaluronic acid. - Large (more than 10 mL of aspirated synovial fluid) effusion in the knee joint or abnormal synovial fluid appearance. - A history of purulent inflammation of the knee. - Rheumatic diseases and systemic diseases of connective tissue. - Active neoplastic disease. - The patient is undergoing oral steroid therapy, antibiotic therapy or biological treatment. - The patient received an intra-articular injection into the examined joint in less than a year before the screening visit. - The patient had previous operations, fractures, ligaments or meniscus injuries in the area of the examined lower limb. - The patient has a Baker’s cyst. - The patient is addicted to nicotine, alcohol, or drugs. - The patient had an injury to the examined knee joint within one month of the screening visit. - The patient has a significant deformation of the examined knee joint. - There is a valgus or varus greater than 10° in any of the knees. - The range of motion of the knee joint is less than 100°. - Major axial deviation (varus > 5, valgus > 5). - Any concomitant symptomatic knee disorder (i.e., ligamentous or meniscal injury). - Hematologic disease. - Active infection. - Recent intra-articular injection of corticosteroid or HA in past 6 months.

**Table 2:** Baseline characteristics of the patients.

		Age	BMI	WOMAC Pain	WOMAC Stiffness	WOMAC Function of the Knee Joint	Total WOMAC
PRP (*n* = 25)	Minimum	40.00	20.30	4.00	0.00	22.00	31.00
	Maximum	70.00	38.10	18.00	8.00	60.00	85.00
	Mean	57.92	27.48	11.28	4.16	38.40	53.84
	Standard Deviation	9.67	4.99	3.34	2.32	10.71	14.96
HA (*n* = 24)	Minimum	40.00	20.80	4.00	0.00	24.00	32.00
	Maximum	66.00	32.20	17.00	8.00	60.00	84.00
	Mean	52.58	26.82	11.88	3.83	39.00	53.92
	Standard Deviation	7.40	3.81	3.62	2.10	10.40	15.19
CS (*n* = 24)	Minimum	46.00	18.21	5.00	0.00	22.00	32.00
	Maximum	69.00	29.75	17.00	8.00	60.00	84.00
	Mean	57.29	25.12	10.33	3.00	38.83	52.17
	Standard Deviation	7.56	3.30	3.32	2.00	9.14	12.89

## Data Availability

The data from this study are available from the corresponding author upon reasonable request.

## Supplementary Materials

The following supporting information can be downloaded at: https://www.mdpi.com/article/10.3390/biomedicines10050991/s1, Figure S1: The gender distribution of Patients.
