# Supplementary figures and images for: Intra-Articular Injection of Platelet-Rich Plasma Is More Effective than Hyaluronic Acid or Steroid Injection in the Treatment of Mild to Moderate Knee Osteoarthritis: A Prospective, Randomized, Triple-Parallel Clinical Trial
Source: Biomedicines. 2022 Apr 25;10(5):991. doi: 10.3390/biomedicines10050991 (PMC9138252; doi:10.3390/biomedicines10050991)

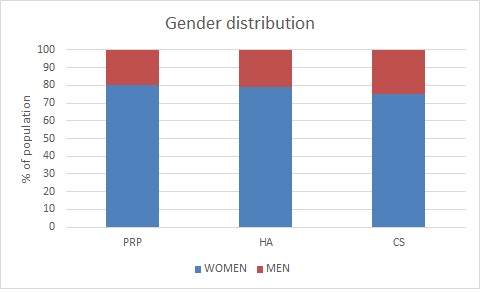

Supplement: Supplementary file 1 [file biomedicines-10-00991-s001.zip › biomedicines-1671504-supplementary.jpg]
